# Hydrazone-based Materials; DFT, TD-DFT, NBO Analysis, Fukui Function, MESP Analysis, and Solar Cell Applications

**DOI:** 10.1007/s10895-022-03000-6

**Published:** 2022-06-23

**Authors:** Mahmoud A. S. Sakr, Farag F. Sherbiny, Abd-Allah Sh. El-Etrawy

**Affiliations:** 1 Department of Chemistry, Center of Basic Science (CBS), Misr University for Science and Technology (MUST), Al-Motamayez District, 6th of the October City 77, Giza, Egypt; 2 Pharmaceutical Organic Chemistry College of Pharmaceutical Science & Drug Manufacturing, Misr University for Science and Technology (MUST), Al-Motamayez District, 6th of the October City 77, Giza, Egypt; 3 Pharmaceutical Organic Chemistry Department, Faculty of Pharmacy (Boys), Al-Azhar University, Cairo, 11884 Egypt

**Keywords:** Quantum calculations, Hydrazone derivatives, Density functional theory (DFT), Natural bond orbital (NBO), TD-DFT

## Abstract

Due to numerous pharmaceutical and biological activities hydrazone (TC) based materials, it was important to investigate quantum chemical studies such as Density functional theory (DFT) calculations, natural bond orbital (NBO) analysis, molecular electrostatic potential (MESP), and local reactivity usage Fukui function for six TC derivatives compounds. DFT, NBO, MESP, and local reactivity calculations were obtained via utilizing CAM-Becke's three-parameter functional and Leee Yange Parr (CAM-B3LYP) functional and 6-311G +  + (2d, 2p) basis set. Optimized molecular structures for all studied compounds were obtained usage the DFT/CAM-B3LYP/6-311G +  + (2d, 2p) method. In addition, the highest occupied molecular orbital (HOMO), lowest unoccupied molecular orbital (LUMO), energy gap (E_g_), light harvest efficiency (LHE), and open-circuit voltage (Voc) of all studied MSs are calculated and illustrated. These properties indicate that these molecular modeling structures as good candidates for utilization in organic DSSCs. The calculated spectroscopic investigations of hydrazine derivatives have been obtained by applying the TD/CAM-B3LYP/6-311G +  + (2d, 2p) method. the calculated UV–Vis absorption spectra for molecular structures under study show nice correlations with experimental spectra.

## Introduction

4-oxo-2-thioxo-1,2,3,4-tetrahydropyrimidine-5-carbohydrazide (TC) based materials are a significant core system and they have gained considerable attention because they are found in various natural and synthetic biologically as well as pharmacologically active compounds [1] due to containing an azomethine moiety (-NH-N = CH-). TC-based materials have been reported to possess a broad spectrum of biological activities including, anticancer [2], antihaemostatic activity [2], antiviral [3], anticonvulsant [4], analgesic [5], anti-inflammatory [6], antiplatelet [9], antifungal [7], and antimalarial activities [8].

Because of the great challenge used in new research on renewable energy sources, solar cells (SCs) are considered one of the most significant renewable energies recently [9–12]. Photovoltaic (PV) technologies have become one of the most important topics in SCs to convert the sun into electrical energy [13, 14]. In addition, the most important challenges are represented in capturing solar energy and converting it into electrical energy at a low cost [15]. It was taken out of the PV devices that are based on inorganic materials in their manufacture, such as crystalline and amorphous silicon (Amorph. Si), cadmium telluride (CdTe), gallium arsenide (GaAs), with the knowledge that they give their efficiency from 10 to 32% [16]. However, since these are expensive materials and scarce, in addition to their toxicity, many researchers have resorted to searching for other new, cheap, and more efficient materials. According to what will be said, SCs based on organic compounds are considered an attractive and appropriate choice due to their flexibility and ease of processing in addition to their low cost, but their efficiency at present time is considered less than those that depend on inorganic materials [17]. The efficiency obtained from these types of cells is still not marketable, as the most efficient devices are 4 to 5% [18].

Recently, new organic compounds used in SCs have been studied and developed. Among these materials are the dyes for sensitive solar cells (DSSCs), as they receive great interest among researchers due to their low cost and high efficiency in converting solar energy into electricity [19]. Moreover, the manufacture of their devices is easy. Also, PV cells based on the DSSCs have many advantages, including their compatibility with many supporting materials and production under moderate conditions that make them less expensive compared to other DSSCs [20, 21]. The first DSSCs were based on titanium dioxide, which was discovered in 1991, and their efficiency was from 7 to 8 percent [13].

In last the study [22], a series of novel *N *^/^-(2-thiouracil-5-oyl) hydrazones were designed, chemically synthesized, and characterized [22]. The anticancer results showed that those compounds exhibit the most prominent effect on breast cancer cells [22]. In addition, molecular docking studies were also performed [22]. Hence, it was important to carry out the following important quantum studies in this manuscript; DFT calculations, NBO analysis, Fukui function analysis, MESP, and solar cell application using CAM-B3LYP/6-311G +  + (2d, 2p) level of theory.

## Studied Molecular Structures (MSs) and Computational Investigation

The molecular modeling and photoelectronic properties of the studied compounds (BTC, CBTC, BBTC, MBTC, MOBTC, and FMTC) were investigated using Density functional theory (DFT) methods via utilizing CAM-B3LYP [23] level with 6-311G +  + (2d, 2p) [24] basis set. All computational calculations were presented via utilizing the Gaussian 09 program [24]. Upon utilizing DFT/CAM-B3LYP/6-31G +  + (2d, 2p) level, the molecular structures (MSs) of neutral molecules are optimized, and their molecular electronic properties as HOMO, LUMO levels, and the energy gap (E_g_) are obtained. The NBO analysis, local reactivity, and electrostatic potential for all studied MSs are calculated via applying the DFT/CAM-B3LYP/6-31G +  + (2d, 2p) level of theory.

**Scheme 1:**
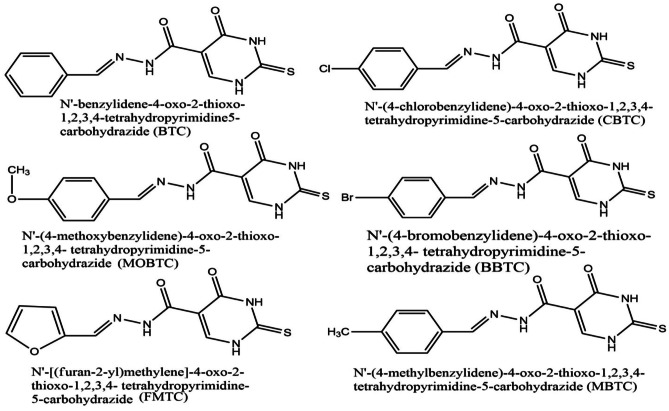
Studied MSs of BTC, CBTC, BBTC, MBTC, MOBTC, and FMTC

All studied MSs(MSs) as shown in Scheme 1 are N'-benzylidene-4-oxo-2-thioxo-1,2,3,4-tetrahydropyrimidine5-carbohydrazide (BTC), N'-(4-chlorobenzylidene)-4-oxo-2-thioxo-1,2,3,4- tetrahydropyrimidine-5-carbohydrazide (CBTC), N'-(4-methoxybenzylidene)-4-oxo-2-thioxo-1,2,3,4- tetrahydropyrimidine-5-carbohydrazide (MOBTC), N'-(4-bromobenzylidene)-4-oxo-2-thioxo-1,2,3,4- tetrahydropyrimidine-5-carbohydrazide (BBTC), N'-[(furan-2-yl)methylene]-4-oxo-2-thioxo-1,2,3,4- tetrahydropyrimidine-5-carbohydrazide and N'-(4-methylbenzylidene)-4-oxo-2-thioxo-1,2,3,4- tetrahydropyrimidine-5-carbohydrazide (MBTC). The six studied MSs under study were prepared, characterized and published in 2021 [22].

## Result and Discussions

### DFT Calculations

**Fig. 1:**
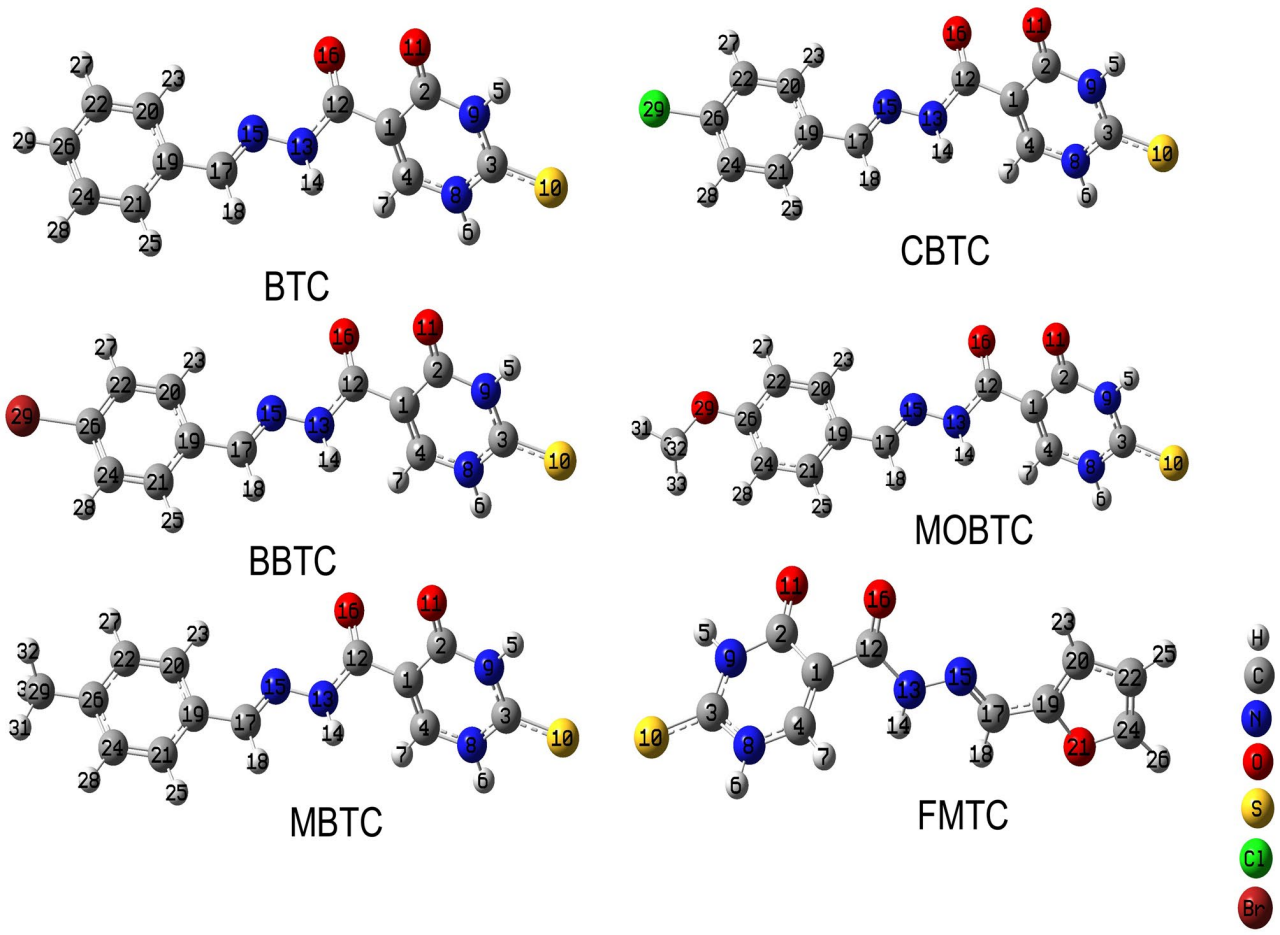
Optimized MSs of BTC, CBTC, BBTC, MBTC, MOBTC, and FMTC in the gaseous state using CAM-B3LYP/6-311G +  + (2d, 2p) level of theory

The optimization of the MSs understudy has been obtained via using DFT/CAM-B3LYP/6-31G +  + (2d, 2p) method in a gaseous state; the results of optimized MSs are presented in Fig. 1. Exitance of C = N in all considered MSs, lead to the possibility of much more than one conformer. The possible conformations for each considered MS were investigated and the more stable conformer for each studied MS is presented in Fig. 1. The influence of substituents such as chloride (Cl) in CBTC, bromide (Br) in BBTC, methoxy (CH_3_-O) in MOBTC, methyl (CH_3_-) in MBTC, and furane in FMTC on some important selected optimized MS parameters such as (bond length in Å, bond angle and dihedral angle in degree) for BTC, CBTC, BBTC, MBTC, MOBTC, and FMTC optimized MSs have been investigated; the results are collected in Table 1. Some important comments that can be deduced are as follows; (i) Referring to the listed values of dihedral angles, all studied MSs are planar. (ii) Owing to increasing bond order, the C_22_-C_20_ bond length is less than C_19_-C_17_ bond lengths in all studied MSs by about (0.016–0.088) Å. (iii) Referring to the listed values of bond angles, the type of hybridization overall studied MSs is SP^2^. (iv) The obtained bond lengths alter because all C–C, ring’s C-N bonds are either doubly bonded or partially multiple bonded. (v) Generally, the stability of MS can be judged via the length of the bond [25], where the more stable molecular structure is combined with the shorter the bond length. As shown in Table 1, the bond lengths of CBTC, BBTC, MBTC, and MOBTC MSs are less than that of the BTC compound. Therefore, the CBTC, BBTC, MBTC, and MOBTC MSs are the highest stable comparable BTC compound. (vi) Also, the bond lengths in FMTC MS are shorter than those in BTC MS. Hence, the FMTC MS is more stable than the BTC compound due to the substitution of furan with the phenyl group.

**Table 1:** Selected optimized structural parameters (bond length in ˚A, bond angle, and dihedral angle in degree computed for BTC, CBTC, BBTC, MBTC, MOBTC, and FMTC in gaseous phase via applying CAM-B3LYP/6-311G +  + (2d, 2p) level of theory. For labeling, belong to Fig. 1

Designation	BTC	CBTC	BBTC	MBTC	MOBTC	FMTC
	Values	Values	Values	Values	Values	Values
C22-C20	1.380	1.378	1.378	1.376	1.370	1.379
C19-C17	1.463	1.462	1.462	1.460	1.458	1.438
N15-N13	1.360	1.354	1.354	1.357	1.359	1.358
C12-O16	1.209	1.197	1.197	1.198	1.198	1.203
C2-O11	1.207	1.200	1.200	1.200	1.201	1.206
C3-S10	1.666	1.651	1.651	1.652	1.652	1.660
C22-C20-C19	120.16	120.44	120.44	120.16	120.54	106.36
C17-N15-N13	116.73	117.58	117.58	117.23	117.11	116.87
N9-C3-N8	114.24	113.6	113.6	113.61	113.62	113.31
C22-C20-C19-C17	179.98	179.99	179.93	179.97	179.97	179.79
C19-C17-N15-N13	179.67	179.39	179.35	179.37	179.35	179.51
C12-C1-C4-N8	175.82	174.03	174.03	174.01	174.01	173.7

**Fig. 2:**
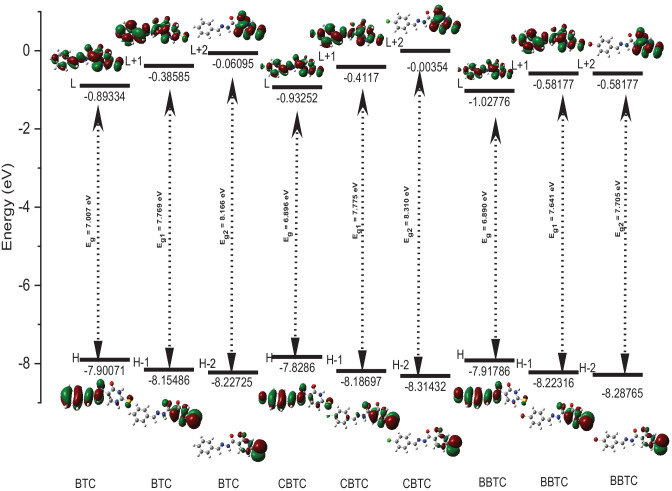
BTC, CBTC, and BBTC molecular modeling graphical presentation of HOMO (H), LUMO (L), H-1, L + 1, H-2, and L + 2 orbitals as well as energy gaps between the following: H/L (E_g_), H-1/L + 1 (E_g1_) and H-2/L + 2 (E_g2_) in the gaseous state via utilizing CAM-B3LYP/6-311G +  + (2d, 2p) level of theory

**Fig. 3:**
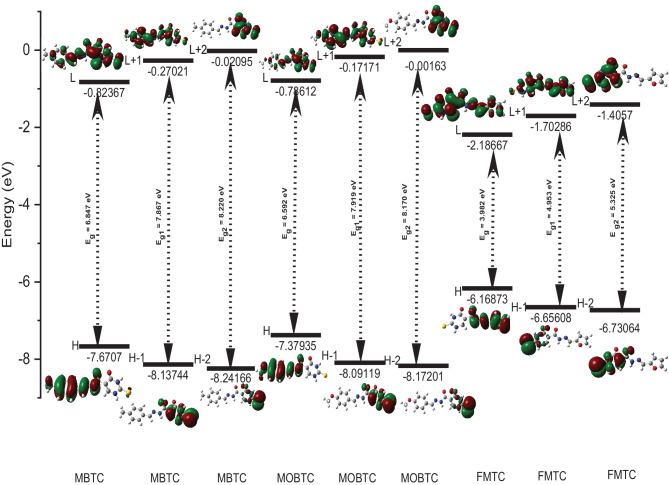
MBTC, MOBTC, and FMTC molecular modeling graphical presentation of HOMO (H), LUMO (L), H-1, L + 1, H-2, and L + 2 orbitals as well as energy gaps between the following: H/L (E_g_), H-1/L + 1 (E_g1_) and H-2/L + 2 (E_g2_) in the gaseous state via utilizing CAM-B3LYP/6-311G +  + (2d, 2p) level of theory

**Fig. 4:**
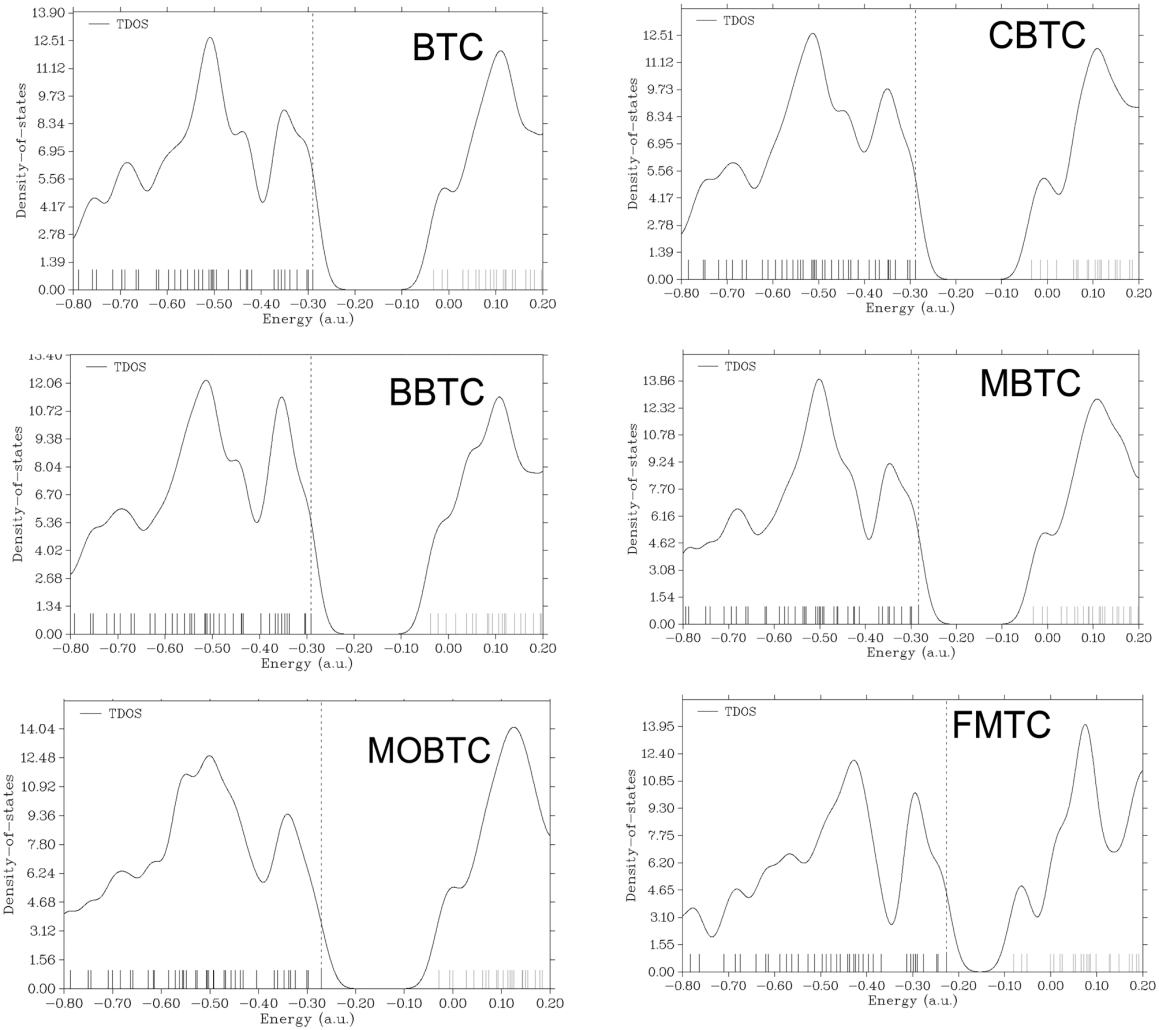
Total density of states (TDOS) spectra for BTC, CBTC, BBTC, MBTC, MOBTC, and FMTC MSs using B3LYP/6-31G* level of theory

The molecular orbitals (MOs) of all studied MSs (BTC, CBTC, BBTC, MBTC, MOBTC, and FMTC) are investigated; the obtained results such as HOMO (H) / LUMO (L), H-1/L + 1, H-2/L + 2 MOs, and energy gaps between the following graphically; H and L (E_g_), H-1 and L + 1 (E_g1_) and H-2 and L + 2 (E_g2_) in the gaseous state via utilizing CAM-B3LYP/6-311G +  + (2d, 2p) level of theory are collected in Figs. 2 and 3. The HOMO MOs are localized on phenyl (-C_6_-H_5_) and its derivatives like (4-Cl-C_6_-H_5_, 4-Br-C_6_-H_5_, 4-CH_3_-C_6_-H_5,_ and 4-CH_3_-O-C_6_-H_5_) in CBTC, BBTC, MBTC, and MOBTC MSs respectively. But, upon substitution of the phenyl group with furan, the HOMO MOs become localized on furan substituent instead of phenyl one. On the other hand, the LUMO MOs are localized over the whole studied MSs. The HOMO energy MOs of all studied MSs are ordered as follow; BBTC < BTC < CBTC < MBTC < MOBTC < FMTC. Indicating, that the BBTC MS has the highest stable HOMO MOs while FMTC MS has the highest reactive HOMO MOs. Also, the LUMO energy MOs are arranged in the following order: FMTC < BBTC < CBTC < BTC < MBTC < MOBTC. This refers to the FMTC MS having the highest stable LUMO MOs, but the MOBTC has the highest reactive LUMO MOs. The energy gap (E_g_) value is calculated by applying the difference (E_L_-E_H_). The calculated E_g_ values for all studied MSs rise in the following order: FMTC < MOBTC < MBTC < BBTC < CBTC < BTC. These results indicate that the FMTC MS is more reactive compared to the other studied MSs and BTC has the lowest reactivity. Also, the reduction in the Eg value favors the red-shifted of the electronic absorption maximum peak in the UV–Vis absorption spectrum. Therefore, the calculated electronic UV–Vis absorption spectra for FMTC, MOBTC, MBTC, BBTC, and CBTC are red shifted, in contrast to, the UV–Vis absorption spectrum of BTC. The total density of states (TDOS) for BTC, CBTC, BBTC, MBTC, MOBTC, and FMTC MSs using the B3LYP/6-31G* level of theory is investigated; the obtained calculated spectra are collected in Fig. 4. The obtained TDOS for all studied MSs are calculated using Multiwfn software [26]. As presented in Fig. 4, the highest reactive MS is FMTC and the lowest one is BTC.

### Quantum Chemical Parameters Calculations

Some important quantum chemical parameters like dipole moment ($\mu$), chemical potential ($\rho$), electronegativity ($\chi$), and chemical hardness ($\eta$) were calculated via using E_L_ and E_H_ values. These quantum parameters are calculated using the following equations $\uprho =\frac{{E}_{H}+ {E}_{L}}{2}$ [27], $\chi=-\frac{E_H+E_L}2\;\mathrm a\mathrm n\mathrm d\;\mathrm\eta=\frac{E_L-E_H}2$ [27]. Generally, when the chemical structure has high dipole moment, it has a large asymmetry in the electric charge distribution, and then it can be highly sensitive due to the change of chemical structure and electronic properties under an external electric field. Thus, as shown in Table 2, the μ value of the compound BTC is highest in contrasting the rest of the other compounds under study. Therefore, this compound is more active compared to the rest of the compounds under study. As presented in Table 2, the ρ value of BBTC MS is lower compared to the other studied compounds. Indicating, that the escaping electrons from BBTC MS are low compared to the rest of the MSs under study. Also, the high $\chi$ value for BBTC MS contrasting the other studied MSs (see Table 2) leads to the ability of this compound to attract electrons from other compounds [28]. On another side, the $\eta$ value for BTC MS is high compared to the rest of the other compounds (see Table 2). This indicates that the BTC compound is exceedingly difficult to liberate electrons, while the other compounds (CBTC, BBTC, MBTC, MOBTC, and FMTC) are good candidates to give electrons to another acceptor molecule.

**Table 2:** Calculated E_H_, E_L_, energy gap (E_g_), dipole moment ($\mu$), and other quantum chemical parameters as electronegativity ($\chi$), chemical potential ($\rho$), chemical hardness ($\eta$) of the BTC, CBTC, BBTC, MBTC, MOBTC and FMTC MSsin gaseous obtained by CAM-B3LYP/6-311G +  + (2d, 2p) level of theory

MSs	E_H_ (eV)	E_L_ (eV)	E_g_(eV)	μ (D)	χ (eV)	ρ (eV)	η (eV)
BTC	-7.900	-0.893	7.007	10.40	4.3965	-4.396	3.503
CBTC	-7.828	-0.932	6.896	6.301	4.380	-4.380	3.448
BBTC	-7.917	-1.027	6.890	6.301	4.472	-4.472	3.445
MBTC	-7.670	-0.823	6.847	7.182	4.246	-4.246	3.423
MOBTC	-7.379	-0.786	6.593	8.489	4.082	-4.082	3.296
FMTC	-6.168	-2.186	3.982	8.016	4.177	-4.177	1.991

### NBO Analysis

The NBO analysis gives an efficient methodology for investigating inter-and intramolecular bonding as well as gives a convenient basis for investigating charge transfer or conjugative interactions in molecular systems [29]. The second-order perturbation energies (stabilization or interaction energies) (E^2^ (Kcal/mol)) and the most significant interaction between Lewis’s type NBOs (donor) and non-Lewis NBOs (acceptor) for all studied MSs are calculated using NBO analysis at CAM-B3LYP/6-311G +  + (2d, 2p) level of theory; the collected data are summarized in Table 3. The results of NBO analysis for BTC, CBTC, BBTC, MBTC, MOBTC, and FMTC MSs indicate that there is a strong hyper conjugative interaction as follows; (1) LP (1) N8 → π*C1-C4 and π*C3-S10, LP (1) N9 → π*C2-O11 and π*C3-S10 as well as LP (1) N13 → π*C12-O16 and π*N15-C17, for all studied MSs (the values of stabilized energy are written in Table 3). Due to furan substituent in FMTC MS, the hyper conjugative energies values for FMTC MS are lowest in contrasting the other studied MSs (BTC, CBTC, BBTC, MBTC, and MOBTC MSs). This indicates the highest reactivity of this compound (FMTC) compared to other studied compounds [29]. As presented in Table 3, the all hyper conjugative interactions values for MBTC and MOBTC are increased compared to BTC MS except πC19-C21 → π*C24-C26, πC20-C22 → π*C19-C21, πC24-C26 → π*C20-C22, LP (1) N8 → π*C1-C4, LP (1) N9 → π*C2-O11, LP (1) N13 → π*N15-C17 and LP (2) O16 → π*C11-N13 hyper conjugative interactions due to electron-donating properties for CH_3_- and CH_3_-O- substituent at the para position of the phenyl group in MBTC and MOBTC MSs respectively. On the other side, the following hyper conjugative interactions for CBTC and BBTC MSs; πC1-C4 → π*C2-O11, πC19-C21 → π*N15-C17, πC20-C22 → π*C19-C21, πC24-C26 → π*C20-C22 and π*C19-C21, LP (1) N8 → π*C3-S10, LP (1) N9 → π*C2-O11 and π*C3-S10, LP (2) O11 → π*C2-N9, LP (1) N13 → π*C12-O16 and LP (2) O16 → π*C1-C12 decreased comparable BTC compound due to electron-withdrawing properties for Cl and Br substituents at the para position of the phenyl group in CBTC and BBTC MSs respectively.

**Table 3:** Selection of most influential second-order perturbation (E^2^) estimation of the hyper conjugative energies (kcal/mol) of the BTC, CBTC, BBTC, MBTC, MOBTC and FMTC molecular modeling structures which were calculated using CAM-B3LYP/6-311G +  + (2d, 2p)) level of theory

Donor	Acceptor	E^2^(Kcal/mol)					
		BTC	CBTC	BBTC	MBTC	MOBTC	FMTC
πC1-C4	π*C2-O11	26.78	26.73	26.73	26.81	26.83	21.67
πC19-C21	π*N15-C17	21.58	21.15	21.07	22.42	23.79	18.79
πC19-C21	π*C20-C22	27.16	27.81	27.61	27.36	28.45	16.79
πC19-C21	π*C24-C26	28.73	30.85	31.09	26.65	23.64	-
πC20-C22	π*C19-C21	30.36	29.11	29.47	28.46	24.71	-
πC20-C22	π*C24-C26	31.32	32.39	32.36	32.04	32.1	-
πC24-C26	π*C20-C22	27.60	26.21	26.52	25.7	21.46	-
πC24-C26	π*C19-C21	31.61	29.04	28.59	33.62	35.01	-
LP (1) N8	π*C1-C4	49.93	50.29	50.31	49.76	49.53	40.25
LP (1) N8	π*C3-S10	80.07	79.63	79.62	80.28	80.54	64.35
LP (1) N9	π*C2-O11	56.02	56.14	56.13	55.97	55.92	45.14
LP (1) N9	π*C3-S10	94.00	93.75	93.79	94.11	94.23	75.71
LP (2) O11	π*C1-C2	23.80	23.93	23.93	23.84	23.81	20.15
LP (2) O11	π*C2-N9	36.64	36.62	36.62	36.65	36.66	31.09
LP (1) N13	π*C12-O16	49.81	49.41	49.41	49.96	50.06	40.21
LP (1) N13	π*N15-C17	29.84	30.75	30.81	29.36	28.78	26.82
LP (2) O16	π*C1-C12	26.33	26.23	26.23	26.36	26.41	22.18
LP (2) O16	π*C11-N13	35.02	35.31	35.33	34.85	34.65	29.48

### Local Reactivity Using Fukui Function

The local reactivity of all atoms in all studied MSs using the Fukui function at the DFT/CAM-B3LYP/6-311G) +  + (2d, 2p) were investigated; the calculated results are written in Tables 4, 5, and 6 [30]. The electrophilic (f^−^), nucleophilic (f^+^) and free radical (f^0^) attack are calculated via utilizing the following equations; f^+^  = [q(N + 1)-q(N)], f^−^ = [q(N)-q(N-1) and f^0^ = [q(N + 1) – q(N-1)]/2 where q is atomic charge (Mulliken, Hirschfeld or NBO, etc.) at the atomic site in the anionic (N + 1), cationic (N-1) or neutral molecule (N). For all studied MSs, the order of the reactive sites for the electrophilic attack, nucleophilic attack, and free radical attacks was collected in Tables 4, 5, and 6. For BTC MS, the parameters of local reactivity descriptors show that 10S is the more reactive site for nucleophilic and free radical attacks and 13 N for electrophilic attacks due to electron-donating (ED) properties of oxygen atom in carbonyl group and nitrogen atom in azomethine moiety (-NH-N = CH-). For BBTC MS, the most reactive site for nucleophilic, free radical, and electrophilic attack is 26C, 16O, and also 16O respectively due to the ED properties of oxygen atoms in the carbonyl group. For FMTC MS, the most reactive site for nucleophilic, free radical, and electrophilic attack is 10S due to ED effects of the oxygen hetero atom in furan substituent. The highest reactive site of electrophilic attack for CBTC MS is 29Cl and the most reactive site for nucleophilic and free radical attack is 10S due to the high electron-withdrawing (EW) properties of chloride substituent. For MOBTC and MBTC MSs, the most reactive electrophilic and nucleophilic attack is the same which is 1C and 10S respectively. But the free radical attack for MOBTC and MBTC MSs is not the same which is 1C for MOBTC and 16O for MBTC. This is due to the highest ED properties for the CH_3_-O group in the MOBTC MS contrasting CH_3_- group in the MBTC compound. (Note: All the compounds under study are compared to the BTC compound, and therefore the reasons for the highest reactive site for nucleophilic, free radical, and electrophilic attacks depend on this comparison).

**Table 4:** Order of the reactive sites on compounds BTC and BBTC

BTC							BBTC						
Atoms	q(N)	q(N + 1)	q(N-1)	f^−^	f^+^	f^0^	Atoms	q(N)	q(N + 1)	q(N-1)	f^−^	f^+^	f^0^
1(C)	-0.046	-0.112	-0.056	0.01	0.0655	0.0277	1(C)	0.0127	-0.036	-0.002	-0.014	-0.023	-0.019
2(C)	0.1746	0.144	0.176	0.0014	0.03	0.0157	2(C)	0.4201	0.4259	0.4175	-0.002	-0.005	-0.004
3(C)	0.111	0.068	0.1124	0.0014	0.0422	0.0218	3(C)	0.4142	0.4455	0.3823	-0.031	-0.031	-0.031
4(C)	0.0433	-0.063	0.0549	0.0115	0.1068	0.0592	4(C)	0.0915	0.0875	0.1159	0.0244	0.0041	0.0142
8(N)	-0.044	-0.060	-0.034	0.0098	0.0156	0.0127	8(N)	-0.315	-0.094	-0.318	-0.002	-0.221	-0.111
9(N)	-0.052	-0.085	-0.048	0.0041	0.0326	0.0183	9(N)	-0.298	-0.282	-0.300	-0.001	-0.016	-0.009
10(S)	-0.282	-0.456	-0.206	0.0761	0.1739	0.1250	10(S)	-0.284	-0.587	-0.237	0.0464	0.3035	0.1749
11(O)	-0.266	-0.326	-0.256	0.0101	0.0597	0.0349	11(O)	-0.377	-0.421	-0.372	0.0054	0.044	0.0247
12(C)	0.1679	0.136	0.1931	0.0253	0.0319	0.0286	12(C)	0.4036	0.0033	0.3916	-0.011	0.4003	0.1942
13(N)	-0.046	-0.049	0.0448	0.0913	0.0033	0.0473	13(N)	-0.230	-0.284	0.0074	0.2380	0.0537	0.1459
15(N)	-0.078	-0.097	-0.005	0.0724	0.0197	0.0461	15(N)	-0.126	-0.125	-0.027	0.0981	-0.000	0.0487
16(O)	-0.264	-0.322	-0.188	0.0757	0.0582	0.0670	16(O)	-0.376	-0.783	-0.338	0.0381	0.4075	0.2228
17(C)	0.027	-0.028	0.0859	0.0589	0.0551	0.057	17(C)	0.115	0.1265	-0.242	-0.357	-0.011	-0.184
19(C)	-0.009	-0.013	0.0424	0.0518	0.0039	0.0279	19(C)	-0.009	0.0134	0.3002	0.3092	-0.022	0.1434
20(C)	-0.028	-0.045	0.0219	0.0501	0.0173	0.0337	20(C)	-0.035	-0.039	0.0407	0.0764	0.0038	0.0401
21(C)	-0.039	-0.059	0.0118	0.0513	0.0195	0.0354	21(C)	-0.018	-0.034	-0.007	0.0107	0.0161	0.0134
22(C)	-0.034	-0.051	0.0039	0.0382	0.0171	0.0276	22(C)	-0.060	-0.043	0.000	0.0602	-0.017	0.0216
24(C)	-0.039	-0.059	0.0059	0.0457	0.0191	0.0324	24(C)	-0.013	0.0311	-0.028	-0.015	-0.044	-0.029
26(C)	-0.033	-0.070	0.0579	0.0912	0.0368	0.064	26(C)	0.0485	0.0423	0.3654	0.3169	0.0062	0.1615
							29(Br)	-0.115	-0.155	-0.046	0.0694	0.0404	0.0549

**Table 5:** Order of the reactive sites on compounds CBTC and MOBTC

CBTC							MOBTC						
Atoms	q(N)	q(N + 1)	q(N-1)	f^−^	f^+^	f^0^	Atoms	q(N)	q(N + 1)	q(N-1)	f^−^	f^+^	f^0^
1(C)	-0.047	-0.095	-0.056	0.0094	0.0479	0.0192	1(C)	-0.046	-0.121	-0.056	0.098	0.0751	0.327
2(C)	0.1746	0.1517	0.1759	0.0013	0.0228	0.0121	2(C)	0.1745	0.1404	0.1752	0.0007	0.0341	0.0174
3(C)	0.1111	0.079	0.1123	0.0012	0.0321	0.0166	3(C)	0.111	0.0632	0.1115	0.0005	0.0478	0.0242
4(C)	0.0438	-0.043	0.0544	0.0106	0.0873	0.0489	4(C)	0.0427	-0.073	0.0516	0.0089	0.1163	0.0626
8(N)	-0.044	-0.058	-0.035	0.009	0.0136	0.0113	8(N)	-0.045	-0.061	-0.038	0.0066	0.0163	0.0115
9(N)	-0.052	-0.077	-0.048	0.0037	0.025	0.0144	9(N)	-0.052	-0.089	-0.050	0.0028	0.0367	0.0198
10(S)	-0.28	-0.426	-0.209	0.0706	0.1466	0.1086	10(S)	-0.285	-0.473	-0.227	0.0577	0.1873	0.1225
11(O)	-0.266	-0.313	-0.257	0.0091	0.0469	0.028	11(O)	-0.267	-0.333	-0.260	0.0065	0.0666	0.0365
12(C)	0.1687	0.1321	0.1918	0.0231	0.0367	0.0299	12(C)	0.1663	0.1387	0.1878	0.0215	0.0276	0.0245
13(N)	-0.045	-0.050	0.0364	0.0824	0.0047	0.0435	13(N)	-0.048	-0.050	0.0146	0.0627	0.0027	0.0327
15(N)	-0.076	-0.113	-0.008	0.068	0.0372	0.0526	15(N)	-0.085	-0.094	-0.014	0.0705	0.0088	0.0396
16(O)	-0.262	-0.322	-0.194	0.0679	0.06	0.064	16(O)	-0.267	-0.322	-0.209	0.0579	0.0551	0.0565
17(C)	0.0262	-0.039	0.0765	0.0503	0.066	0.0581	17(C)	0.0256	-0.021	0.0565	0.0308	0.0475	0.0392
19(C)	-0.008	-0.019	0.0445	0.0531	0.0109	0.032	19(C)	-0.024	-0.022	0.0467	0.0707	-0.001	0.0345
20(C)	-0.020	-0.045	0.0238	0.0447	0.0246	0.0347	20(C)	-0.023	-0.034	0.014	0.0378	0.0109	0.0244
21(C)	-0.032	-0.057	0.0152	0.0479	0.0252	0.0365	21(C)	-0.036	-0.053	0.015	0.0516	0.017	0.0343
22(C)	-0.037	-0.057	-0.002	0.035	0.0192	0.0271	22(C)	-0.050	-0.063	-0.001	0.0491	0.0128	0.0309
24(C)	-0.043	-0.065	-0.001	0.0424	0.0222	0.0323	24(C)	-0.070	-0.082	-0.015	0.0544	0.0127	0.0335
26(C)	0.0292	-0.008	0.0961	0.0669	0.0373	0.0521	26(C)	0.0824	0.0585	0.1505	0.0681	0.0239	0.046
29(Cl)	-0.056	-0.116	0.0779	0.1345	0.0603	0.0974	29(O)	-0.125	-0.139	-0.050	0.0751	0.014	0.0445
							30(C)	0.0046	-0.001	0.0253	0.0207	0.0064	0.0135

**Table 6:** Order of the reactive sites on compounds MBTC and FMTC

MBTC							FMTC						
Atoms	q(N)	q(N + 1)	q(N-1)	f^−^	f^+^	f^0^	Atoms	q(N)	q(N + 1)	q(N-1)	f^−^	f^+^	f^0^
1(C	-0.046	-0.115	-0.057	0.105	0.0683	0.0289	1(C)	-0.044	-0.091	0.0373	0.0067	0.0479	0.0273
2(C)	0.1745	0.1433	0.1755	0.001	0.0312	0.0161	2(C)	0.1676	0.1445	0.1764	0.0087	0.0231	0.0159
3(C)	0.111	0.0672	0.1118	0.0008	0.0438	0.0223	3(C)	0.1022	0.0623	0.1195	0.0173	0.0399	0.0286
4(C)	0.0431	-0.066	0.0534	0.0103	0.1095	0.0599	4(C)	0.0399	-0.039	0.0545	0.0146	0.0789	0.0467
8(N)	-0.044	-0.060	-0.036	0.0081	0.0158	0.0119	8(N)	-0.040	-0.057	-0.024	0.0155	0.0168	0.0162
9(N)	-0.052	-0.086	-0.049	0.0033	0.0338	0.0185	9(N)	-0.050	-0.079	-0.036	0.0136	0.0287	0.0211
10(S)	-0.284	-0.461	-0.218	0.066	0.1774	0.1217	10(S)	-0.272	-0.433	-0.074	0.1982	0.1614	0.1798
11(O)	-0.266	-0.328	-0.258	0.0082	0.0617	0.0349	11(O)	-0.263	-0.312	-0.234	0.0292	0.0485	0.0388
12(C)	0.1672	0.1367	0.1912	0.024	0.0305	0.0272	12(C)	0.1624	0.1298	0.1822	0.0198	0.0325	0.0262
13(N)	-0.047	-0.050	0.0326	0.0798	0.003	0.0414	13(N)	-0.038	-0.047	0.0136	0.052	0.0091	0.0306
15(N)	-0.081	-0.097	-0.008	0.0724	0.016	0.0442	15(N)	-0.084	-0.123	-0.031	0.0534	0.0392	0.0463
16(O)	-0.266	-0.323	-0.197	0.0691	0.057	0.0631	16(O)	-0.26	-0.317	-0.206	0.0532	0.0578	0.0555
17(C)	0.0268	-0.026	0.0743	0.0475	0.0527	0.0501	17(C)	0.0161	-0.048	0.0535	0.0375	0.0647	0.0511
19(C)	-0.014	-0.016	0.0454	0.06	0.0015	0.0308	19(C)	0.0477	0.0342	0.1029	0.0552	0.0135	0.0344
20(C)	-0.028	-0.042	0.0177	0.0462	0.0144	0.0303	20(C)	-0.053	-0.1	0.0093	0.0632	0.0461	0.0546
21(C)	-0.039	-0.058	0.0121	0.052	0.0189	0.0354	21(O)	-0.08	-0.103	-0.050	0.0296	0.0233	0.0264
22(C)	-0.040	-0.054	-0.001	0.0384	0.014	0.0262	22(C)	-0.066	-0.095	-0.012	0.0545	0.0289	0.0417
24(C)	-0.047	-0.062	-0.000	0.0465	0.0154	0.031	24(C)	0.0277	-0.023	0.1192	0.0916	0.0511	0.0713
26(C)	0.0135	-0.015	0.0934	0.0799	0.0286	0.0542							
29(C)	-0.082	-0.090	-0.059	0.0225	0.0087	0.0156							

### Molecular Electrostatic Potential (MESP)

**Fig. 5:**
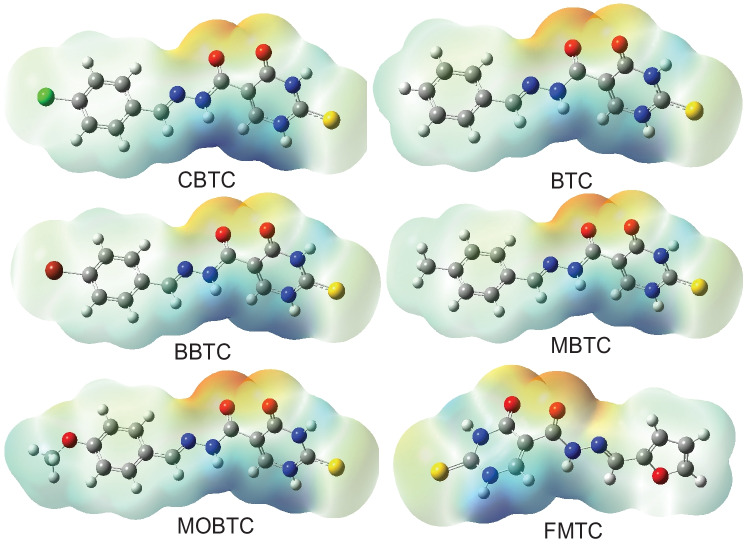
Calculated MESP on the molecular surfaces of studied compounds.CAM-B3LYP functional and 6-311G +  + (2d, 2p) basis set

The electron density is an incredibly significant factor for investigating the reactivity of electrophilic (E) and nucleophilic (Nu) sites and the interactions of hydrogen bonds [30], as well as this density, is related to the molecular electrostatic potential (MESP). Therefore, for predicting this reactivity of Nu and E sites attacks for all studied MSs, we obtained the MESP of these MSs usage the CAM-B3LYP /6-311G +  + (2d, 2p) level of theory. The numerous colors (red, blue, and green) at the MESP surface indicate different values of the ESP as the regions of highest negative, highest positive, and zero ESP respectively. The negative sites at MESP (red) refer to E reactivity, the positive sites (blue) refer to Nu reactivity, and the green represents regions of zero potential (See Fig. 5). As presented in Fig. 5, the maximum positive region for all studied MSs is localized on a hydrogen atom and is linked to a nitrogen atom (N–H) which indicates that this area is an attracting site of electrons. On the other side, the maximum negative region for all studied MSs is localized on the carbonyl group (C = O) which indicates this area is a donating site of electrons.

### Application

**Fig. 6:**
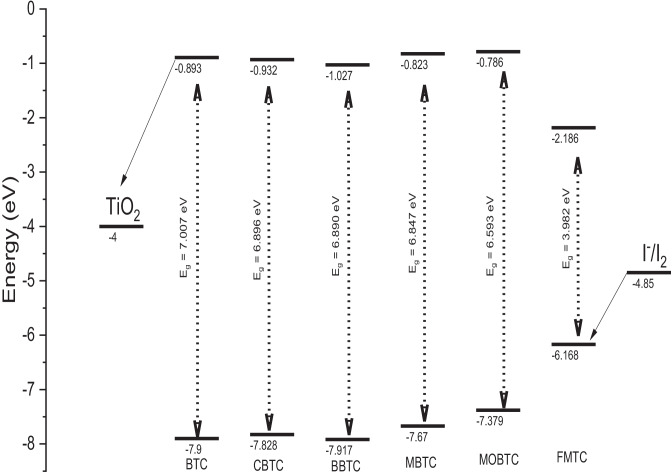
Frontier molecular orbital energies and energy gaps of TiO_2_, BTC, CBTC, BBTC, MBTC, MOBTC, FMTC, and I.^−^/I_2_

Figure 6 exhibits the MO energy levels of the six MSs BTC, CBTC, BBTC, MBTC, MOBTC, and FMTC in a gaseous state at the DFT/CAM-B3LYB/6-31G +  + (2d, 2p) level of theory. For the MSs understudy, the HOMO energy is lower than the energy of I^−^/I_2_ (− 4.85 eV). This indicates that all studied MSs (BTC, CBTC, BBTC, MBTC, MOBTC, and FMTC) can more easily recover electrons from electrolytes (I_3_^−^). In addition, the LUMO energy of the six studied molecules (BTC, CBTC, BBTC, MBTC, MOBTC, and FMTC) is higher than the conduction band (CB) energy of semiconductor TiO_2_ (− 4.00 eV) as shown in Fig. 6. Indicating, that the electrons can be successfully transferred into TiO_2_ from the excited state of all studied dyes. Consequently**,** all studied MSs dyes may be good candidates for application in PV devices.

The conversion efficiency (Ƞ) of sunlight to electrical energy in SC devices is determined by the short-circuit current density (*J*sc), the open-circuit PV (Voc), the fill factor (FF), and incident solar power (Pinc). The Ƞ can be estimated via utilizing Eq. (1) [31]:

1 $$\eta =\frac{{J}_{sc } {V}_{oc} FF}{{P}_{inc}}$$

from the formula, it can be seen that high V_OC_ and J_SC_ are the basis for producing photoelectric conversion efficiency. The maximum value for Voc is a significant PV parameter that can be obtained computationally via utilizing the difference between the HOMO of dye and the LUMO of the electron acceptor (conduction band of TiO_2_). The computational value of Voc has been calculated via utilizing Eq. (2) [27, 32]:

2 $${V}_{oc}=\left|{E}_{HOMO}^{donor}\right|-\left|{E}_{LUMO}^{acceptor}\right|-0.3$$

However, in DSSCs, Voc can be obtained as the different energy among LUMO of the dye and conduction band (CB) of the semiconductor [27, 32].

3 $${{TiO}_2\;\left(E_{CB}=-4.0\;eV\right):V}_{oc}=\left|E_{LUMO}^{dye}\right|-E_{CB}$$

The theoretically calculated value of Voc for all studied MSs is presented in Table 7. The range value of Voc is 1.814 to 3.214 eV for TiO_2_. These values are positive; indicating the electron transfer will be easy from all studied MSs to TiO_2_. Furthermore, these values are sufficient to give the best efficient electron injection. Moreover, those studied MSs can be utilized as sensitizers of the electron injection process from the excited dye to the conduction band of TiO_2_.

**Table 7:** Energy values of E_HOMO_, E_LUMO_, open-circuit voltage (Voc), and light-harvesting efficiency (LHE) by eV

Compounds	E_HOMO_ (eV)	E_LUMO_ (eV)	Voc (eV)	α (eV)	LHE (ev)
BTC	-7.900	-0.893	3.107	2.307	0.856
CBTC	-7.828	-0.932	3.068	2.268	0.944
BBTC	-7.917	-1.027	2.973	2.173	0.951
MBTC	-7.670	-0.823	3.177	2.377	0.934
MOBTC	-7.379	-0.786	3.214	2.414	0.944
FMTC	-6.168	-2.186	1.814	1.014	0.636

Another parameter denoted (α) was calculated via the difference between the LUMO energy levels of the studied dyes and the LUMO energy level of PCBM (-3.2 eV) [33]. The value can be obtained via using Eq. (4):

4 $$\alpha =\left|{E}_{LUMO}^{acceptor}\right|- \left|{E}_{LUMO}^{donor}\right|$$

The light-harvesting efficiency (LHE) is obtained using the following equation (LHE = 1–10^−*f*^) [33], where *f* is the oscillator strength of the dye MS; the calculated LHE is presented in Table 7. The *f* values for all studied MSs were calculated using the TD-DFT/CAM-B3LYP/6-3111G +  + (2d, 2p) method.

As presented in Table 7, the obtained values of α were in the range of (1.014–2.414 eV). Indicating, that all LUMO MO level for all studied MSs is placed higher than the LUMO MO level of PCBM [33]. So, these studied compounds can be utilized as sensitizers being of the electron injection process from the excited dye to the conduction band of PCBM. Since the value of LHE increase in the following order as shown in Table 7; FMTC < BTC < MBTC < CBTC = MOBTC < BBTC therefore the best dye that can act as DSSCs is BBTC dye comparable to the rest compounds under study. The LHE value for BTC was 0.856 eV as shown in Table 7. The presence of chloride (Cl^−^), bromide (Br^−^), methyl (CH_3_-), and methoxy (CH_3_-O-) substituents at the para position of the phenyl group in CBTC, BBTC, MBTC, and MOBTC MSs respectively enhance the LHE by the range of (0.078–0.095) eV. This is due to the EW properties of Cl^−^ and Br^−^ and ED properties of CH_3_- and CH_3_-O-. On the other hand, comparable FMTC to BTC, the LHE of FMTC is decreased via 0.22 eV due to the substitution of furan with a phenyl group. The LHE values for BTC, CBTC, BBTC, MBTC, and MOBTC were in the close range (0.856–0.951) indicating that those MSs gave similar photocurrent [34].

### Spectroscopic Investigations

**Fig. 7:**
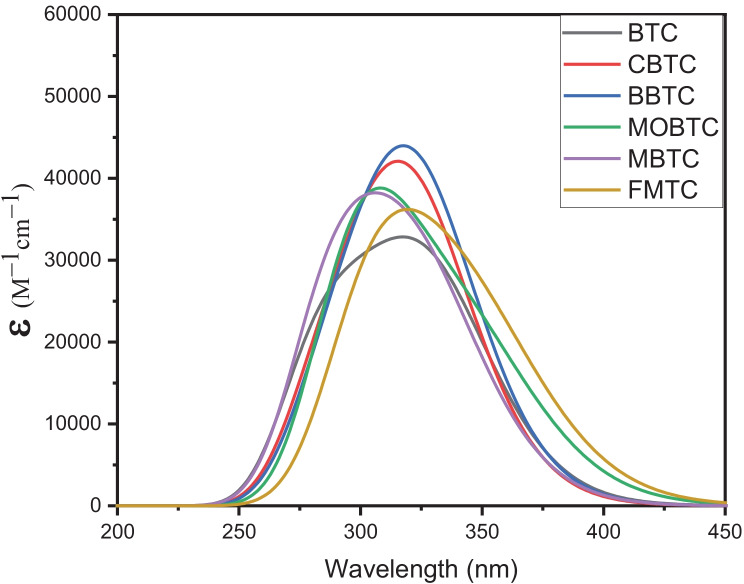
Calculated electronic absorption spectra for BTC, CBTC, BBTC, MOBTC, MBTC, and FMTC MSs using TD/CAM-B3LYP/6-311G +  + (2d, 2p) method

The experimental absorption spectra for hydrazone-based materials class were discussed in the past [35, 36]. Those spectra were within the 300–450 nm range, and the reason for those electronic absorption spectra was π-π* electronic transition [36]. The accurate functional used to calculate the electronic UV–Vis absorption spectra for the hydrazone derivatives family was B3LYP [36]. But the reviewer recommended using CAM-B3LYP instead of B3LYP as the density functional B3LYP is well known to poorly handle dispersion interactions. Hence, the computational electronic UV–Vis absorption spectra for TC-based materials in methanol were calculated using the TD/CAM-B3LYP/6-311G +  + (2d, 2p) method; the results calculated spectra are presented in Fig. 7 and the corresponding optical parameters are written in Table 8. The computational UV–Vis absorption spectra for all studied MSs are within 250-450 nm rang as shown in Fig. 7. Comparing the computational results with the experimental one, it was shown that the computational electronic UV–Vis absorption spectra for all studied MSs lie in the same region as the experimental one. Therefore, the collected computational results are nice with the experimental. The calculated electronic absorption spectrum of BTC, CBTC, BBTC, MBTC, MOBTC, and FMTC MSs in methanol appears as three transitions. The electronic transitions for BTC at 314.71, 286.45 and 267.39 nm, for CBTC at 311.10, 285.34 and 266.33 nm, for BBTC at 305.18, 261.29 and 261.29 nm, for MBTC at 304.43, 278.38 and 260.09 for MOBTC at 304.02, 284.24 and 261.64 nm and for FMTC at 307.52, 294.06 and 264.83 nm. The electronic transitions referring to the calculated maximum peak at 286.45 nm (f = 1.2728) for BTC, 266.33 nm (f = 0.3550) for CBTC, 261.29 nm (f = 1.5791) for BBTC, for MBTC, 278.38 nm (f = 1.4876) for MOBTC and 294.06 nm(f = 1.2591) For FMTC respectively arises from transition of MO71—> 72, MO79—> 80, MO88—> 89, MO75—> 76, MO74—> 76 and MO78—> 79 respectively as presented in Table 8.

**Table 8:** Calculated electronic absorption of hydrazone-based materials using TD/CAM-B3LYP/6-311G +  + (2d, 2p)

TD-Computational				
BTC				
Excited state	Electronic transitions	ΔE (eV)	f	Coefficient
1	69- > 72 69—> 73	3.939 (314.71 nm)	0.0008	0.1553 0.8812
2	69—> 74 70—> 72 70—> 73 71—> 72	4.328 (286.45 nm)	1.2728	0.0233 0.1311 0.7327 0.0236
3	71—> 73 70—> 72 71—> 72 71—> 73	4.636 (267.39 nm)	0.1041	0.8403 0.0470 0.0330 0.0243
CBTC				
1	77—> 80 77—> 81	3.985 (311.10 nm)	0.0005	0.0543 0.8881
2	77—> 82 77—> 81 77—> 82 78—> 80 78—> 81 79—> 80	4.345 (285.34 nm)	0.0945	0.7889 0.0486 0.0306 0.0220 0.0246 0.0525
3	79—> 81 78—> 80 78—> 81 79—> 80	4.655 (266.33 nm)	0.3559	0.0256 0.0404 0.8200 0.0469
BBTC				
1	86—> 89 86—> 90	4.062 (305.18 nm)	0.0005	0.0261 0.8905
2	86—> 91 87—> 89 87—> 90 88—> 89	4.745 (261.29 nm)	1.5791	0.0278 0.0321 0.8477 0.0355
3	88—> 90 87—> 89 87—> 90	4.745 (261.29 nm)	0.0736	0.7930 0.0472 0.0299
MBTC				
1	73—> 76 73—> 77	4.072 (304.43 nm)	0.0005	0.4120 0.8820
2	74—> 76 74—> 77	4.453 (278.38 nm)	1.4876	0.6202 0.1401
3	74—> 76 74—> 77	4.766 (260.09 nm)	0.0749	0.1789 0.1659
MOBTC				
1	77—> 80 77—> 81	4.0781 (304.02 nm)	0.0009	0.0225 0.9450
2	77—> 82 78—> 80	4.362 (284.24 nm)	1.4676	0.3366 0.6072
3	78—> 80 78—> 80	4.7388 (261.64)	0.1225	0.0273 0.0917
FMTC				
1	66—> 79 66—> 60	4.0318 (307.52 nm)	0.0019	0.0275 0.9113
2	66—> 71 67—> 69 67—> 70	4.2163 (294.06 nm)	1.2591	0.3248 0.6066 0.0200
3	68—> 69 67—> 69 67—> 70	4.681 (264.83 nm)	0.1836	0.5235 0.0304 0.0544

## Conclusion

In this paper, the optimized molecular structures, NBO analysis, ESP calculation, Fukui function analysis, and DFT calculations of six 1,2,3,4-tetrahydropyrimidine-5-carbohydrazide (BTC, CBTC, BBTC, MBTC, MOBTC, and FMTC) were investigated successfully via utilizing DFT/CAM-B3LYP/6-311G +  + (2d, 2p) method. According to the ground state geometry, we note that all stable conformations are planar. The HOMO/LUMO energy gaps of BTC, CBTC, BBTC, MBTC, MOBTC, and FMTC were calculated at DFT/CAM-B3LYP/6-31G +  + (2d, 2p) levels are 7.007, 6.896, 6.890, 6.847, 6.593 and 3.982 eV respectively. So, the energy gaps differ slightly and decrease in the following order: FMTC < MOBTC < MBTC < BBTC < CBTC < BTC. Consequently, the calculated values of Voc/ TiO_2_ of our dyes are sufficient for possible efficient electron injection from the donor to the acceptor.

## Data Availability

All data generated or analyzed during this study are included in this published article.
